# 4-Methyl­pyridinium 4-hy­droxy­benzoate

**DOI:** 10.1107/S1600536813001785

**Published:** 2013-01-23

**Authors:** S. Sudhahar, M. Krishnakumar, B. M. Sornamurthy, G. Chakkaravarthi, R. Mohankumar

**Affiliations:** a Department of Physics, Presidency College, Chennai 600 005, India; b Department of Physics, CPCL Polytechnic College, Chennai 600 068, India

## Abstract

In the crystal structure of the title salt, C_6_H_8_N^+^·C_7_H_5_O_3_^−^, the anions and cations are linked by classical N—H⋯O hydrogen bonds. The anions are connected by pairs of C—H⋯O hydrogen bonds into inversion dimers and further linked by classical O—H⋯O hydrogen bonds. Weak π–π inter­actions [centroid–centroid distances = 3.740 (3) and 3.855 (3) Å] also occur. The dihedral angle between the CO_2_^−^ group and the benzene ring to which it is attached is 20.95 (8)°.

## Related literature

For biological applications of picolinium-containing compounds, see: Butler & Walker (1993 ▶); Roy *et al.* (2001 ▶). For bond-length data, see: Allen *et al.* (1987 ▶).
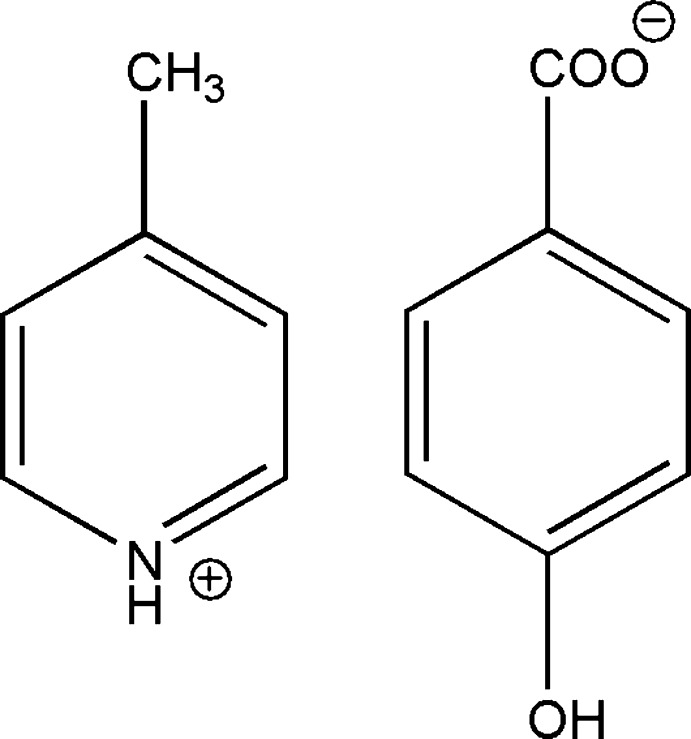


## Experimental

### 

#### Crystal data

C_6_H_8_N^+^·C_7_H_5_O_3_^−^
*M* *_r_* = 231.24
Monoclinic, 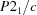

*a* = 7.479 (5) Å
*b* = 11.671 (4) Å
*c* = 13.520 (5) Å
β = 100.217 (5)°
*V* = 1161.4 (10) Å^3^
*Z* = 4
Mo *K*α radiation
μ = 0.10 mm^−1^
*T* = 295 K
0.24 × 0.20 × 0.18 mm

#### Data collection

Bruker Kappa APEXII CCD diffractometer
Absorption correction: multi-scan (*SADABS*; Sheldrick, 1996 ▶) *T*_min_ = 0.978, *T*_max_ = 0.983
11741 measured reflections
2564 independent reflections
1939 reflections with *I* > 2σ(*I*)
*R*_int_ = 0.029

#### Refinement

*R*[*F* ^2^ > 2σ(*F* ^2^)] = 0.043
*wR*(*F* ^2^) = 0.128
*S* = 1.06
2564 reflections
156 parameters
H-atom parameters constrained
Δρ_max_ = 0.38 e Å^−3^
Δρ_min_ = −0.34 e Å^−3^

### 

Data collection: *APEX2* (Bruker, 2004 ▶); cell refinement: *SAINT* (Bruker, 2004 ▶); data reduction: *SAINT*; program(s) used to solve structure: *SHELXS97* (Sheldrick, 2008 ▶); program(s) used to refine structure: *SHELXL97* (Sheldrick, 2008 ▶); molecular graphics: *PLATON* (Spek, 2009 ▶); software used to prepare material for publication: *SHELXL97*.

## Supplementary Material

Crystal structure: contains datablock(s) I, global. DOI: 10.1107/S1600536813001785/rk2392sup1.cif

Structure factors: contains datablock(s) I. DOI: 10.1107/S1600536813001785/rk2392Isup2.hkl

Supplementary material file. DOI: 10.1107/S1600536813001785/rk2392Isup3.cml

Additional supplementary materials:  crystallographic information; 3D view; checkCIF report

## Figures and Tables

**Table 1:** Hydrogen-bond geometry (Å, °)

*D*—H⋯*A*	*D*—H	H⋯*A*	*D*⋯*A*	*D*—H⋯*A*
O1—H1⋯O2^i^	0.82	1.85	2.6707 (19)	176
N1—H1*A*⋯O3^ii^	0.86	1.73	2.5889 (19)	173
C2—H2⋯O1^iii^	0.93	2.60	3.485 (2)	160

Symmetry codes: (i) 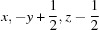; (ii) 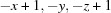; (iii) 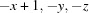.

**Fig. 1.**
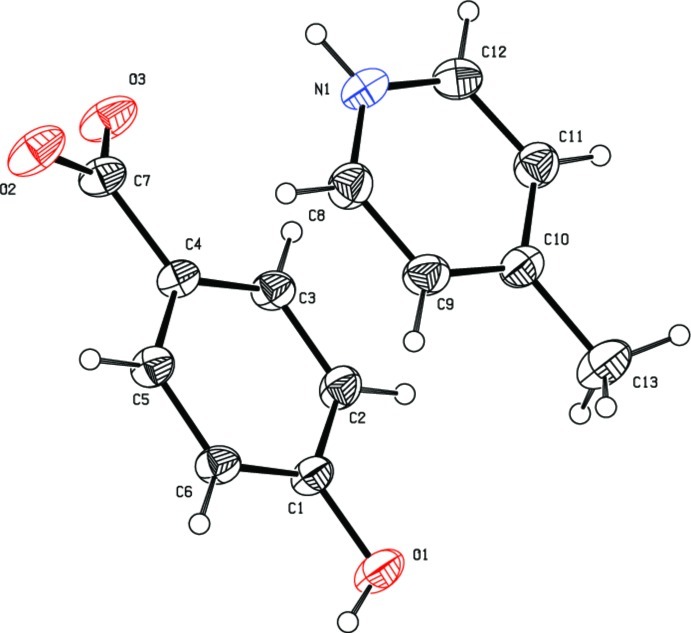
The molecular structure of title compound with the atom numbering scheme. Displacement ellipsoids are drawn at 30% probability level. H atoms are presented as a small spheres of arbitrary radius.

**Fig. 2.**
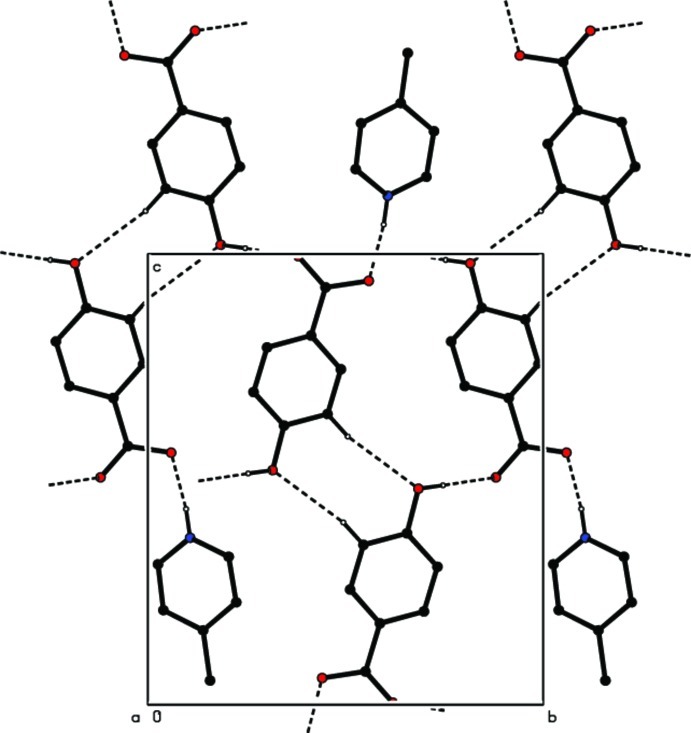
The crystal packing of I, viewed down a axis. Intermolecular hydrogen bonds are shown as dashed lines. H atoms not involved in hydrogen bonding have been omitted for clarity.

## supplementary crystallographic information

### Comment

Picolinium compounds are valuable intermediates in organic synthesis and they have been used widely in industrially important products and biologically active substrates as antitumor, antifungal, antibacterial, antineoplastic and antviral (Butler & Walker, 1993; Roy *et al.*, 2001) activities.

The asymmetric unit of the title salt, I, (Fig. 1), contains C_6_H_8_N^+^ cation and C_7_H_5_O_3_^-^ anion. The bond lengths and angles in both anion and cation are within normal range (Allen *et al.*, 1987). The crystal structure exhibit weak intermolecular classical N—H···O, O—H···O and non–classical C—H···O interactions (Table 1 & Fig. 2). The π–π interactions are found in crystal structure: *Cg*1···*Cg*2^iv^ = 3.740 (3)Å; *Cg*1···*Cg*2^v^ = 3.855 (3)Å, where *Cg*1 and *Cg*2 are the centroids of the rings (C1–C6) and (N1/C8–C12), respectively. Symmetry codes: (iv) *x*, -*y*+1/2, *z*+1/2); (v) *x*+1, *y*, *z*;

### Experimental

4-Picolinium 4-hydroxybenzoate compound was synthesized by using the starting materials of 4-picoline (1.66 g) and 4-hydroxybenzoic acid (1.12 g) in methanol and the single crystals suitable for X-ray diffraction were grown by slow evaporation.

### Refinement

The H atoms were positioned geometrically with C—H = 0.93Å and 0.96Å, O—H = 0.82Å and N—H = 0.86Å, and allowed to ride on their parent atoms, with *U*_iso_(H) = 1.5*U*_eq_(O) for hydroxy group, *U*_iso_(H) = 1.2*U*_eq_(N) for amino group, *U*_iso_(H) = 1.2*U*_eq_(C) for aryl H and *U*_iso_(H) = 1.5*U*_eq_(C) for methyl H.

### Crystal data

**Table:**

C_6_H_8_N^+^·C_7_H_5_O_3_^−^	*F*(000) = 488
*M**_r_* = 231.24	*D*_x_ = 1.322 Mg m^−^^3^
Monoclinic, *P*2_1_/*c*	Melting point = 470.4–481.2 K
Hall symbol: -P 2ybc	Mo *K*α radiation, λ = 0.71073 Å
*a* = 7.479 (5) Å	Cell parameters from 7082 reflections
*b* = 11.671 (4) Å	θ = 2.3–27.1°
*c* = 13.520 (5) Å	µ = 0.10 mm^−^^1^
β = 100.217 (5)°	*T* = 295 K
*V* = 1161.4 (10) Å^3^	Block, colourless
*Z* = 4	0.24 × 0.20 × 0.18 mm

### Data collection

**Table:**

Bruker Kappa APEXII CCD diffractometer	2564 independent reflections
Radiation source: fine–focus sealed tube	1939 reflections with *I* > 2σ(*I*)
Graphite monochromator	*R*_int_ = 0.029
ω and φ scans	θ_max_ = 27.2°, θ_min_ = 2.3°
Absorption correction: multi-scan (*SADABS*; Sheldrick, 1996)	*h* = −9→9
*T*_min_ = 0.978, *T*_max_ = 0.983	*k* = −14→8
11741 measured reflections	*l* = −17→17

### Refinement

**Table:**

Refinement on *F*^2^	Secondary atom site location: difference Fourier map
Least-squares matrix: full	Hydrogen site location: inferred from neighbouring sites
*R*[*F*^2^ > 2σ(*F*^2^)] = 0.043	H-atom parameters constrained
*wR*(*F*^2^) = 0.128	*w* = 1/[σ^2^(*F*_o_^2^) + (0.058*P*)^2^ + 0.3003*P*] where *P* = (*F*_o_^2^ + 2*F*_c_^2^)/3
*S* = 1.06	(Δ/σ)_max_ < 0.001
2564 reflections	Δρ_max_ = 0.38 e Å^−^^3^
156 parameters	Δρ_min_ = −0.34 e Å^−^^3^
0 restraints	Extinction correction: *SHELXL97* (Sheldrick, 2008), Fc^*^=kFc[1+0.001xFc^2^λ^3^/sin(2θ)]^-1/4^
Primary atom site location: structure-invariant direct methods	Extinction coefficient: 0.010 (2)

### Special details

**Table:**

Geometry. All s.u.'s (except the s.u. in the dihedral angle between two l.s. planes) are estimated using the full covariance matrix. The cell s.u.'s are taken into account individually in the estimation of s.u.'s in distances, angles and torsion angles; correlations between s.u.'s in cell parameters are only used when they are defined by crystal symmetry. An approximate (isotropic) treatment of cell s.u.'s is used for estimating s.u.'s involving l.s. planes.
Refinement. Refinement of *F*^2^ against ALL reflections. The weighted *R*–factor *wR* and goodness of fit *S* are based on *F*^2^, conventional *R*–factors *R* are based on *F*, with *F* set to zero for negative *F*^2^. The threshold expression of *F*^2^ > σ(*F*^2^) is used only for calculating *R*–factors(gt) *etc*. and is not relevant to the choice of reflections for refinement. *R*–factors based on *F*^2^ are statistically about twice as large as those based on *F*, and *R*–factors based on ALL data will be even larger.

### Fractional atomic coordinates and isotropic or equivalent isotropic displacement parameters (Å^2^)

**Table:**

	*x*	*y*	*z*	*U*_iso_*/*U*_eq_	
O1	0.55020 (18)	0.18454 (10)	0.02055 (8)	0.0578 (4)	
H1	0.5981	0.2464	0.0131	0.087*	
O2	0.7215 (2)	0.11967 (10)	0.49538 (9)	0.0599 (4)	
O3	0.71511 (18)	−0.05893 (9)	0.44053 (8)	0.0563 (4)	
C1	0.5832 (2)	0.15548 (13)	0.11952 (11)	0.0396 (4)	
C2	0.5407 (2)	0.04549 (13)	0.14510 (11)	0.0435 (4)	
H2	0.4883	−0.0056	0.0955	0.052*	
C3	0.5761 (2)	0.01174 (12)	0.24435 (11)	0.0394 (4)	
H3	0.5492	−0.0628	0.2612	0.047*	
C4	0.6512 (2)	0.08716 (12)	0.31929 (10)	0.0356 (3)	
C5	0.6894 (2)	0.19833 (12)	0.29287 (11)	0.0394 (4)	
H5	0.7375	0.2503	0.3427	0.047*	
C6	0.6571 (2)	0.23261 (13)	0.19390 (11)	0.0397 (4)	
H6	0.6846	0.3070	0.1769	0.048*	
C7	0.6972 (2)	0.05049 (13)	0.42610 (11)	0.0412 (4)	
N1	0.18709 (19)	0.10699 (12)	0.37067 (10)	0.0475 (4)	
H1A	0.2170	0.0966	0.4344	0.057*	
C8	0.2266 (2)	0.20451 (14)	0.32853 (13)	0.0490 (4)	
H8	0.2872	0.2617	0.3692	0.059*	
C9	0.1813 (2)	0.22368 (14)	0.22732 (12)	0.0473 (4)	
H9	0.2099	0.2933	0.2005	0.057*	
C10	0.0932 (2)	0.13982 (14)	0.16510 (11)	0.0446 (4)	
C11	0.0517 (2)	0.03949 (14)	0.20988 (13)	0.0480 (4)	
H11	−0.0091	−0.0190	0.1709	0.058*	
C12	0.1000 (2)	0.02581 (14)	0.31174 (13)	0.0478 (4)	
H12	0.0710	−0.0424	0.3407	0.057*	
C13	0.0475 (3)	0.15746 (19)	0.05391 (14)	0.0686 (6)	
H13A	0.0259	0.2374	0.0398	0.103*	
H13B	−0.0596	0.1145	0.0272	0.103*	
H13C	0.1469	0.1318	0.0234	0.103*	

### Atomic displacement parameters (Å^2^)

**Table:**

	*U*^11^	*U*^22^	*U*^33^	*U*^12^	*U*^13^	*U*^23^
O1	0.0913 (9)	0.0446 (7)	0.0328 (6)	−0.0132 (6)	−0.0014 (6)	0.0060 (5)
O2	0.1019 (10)	0.0430 (7)	0.0334 (6)	0.0124 (6)	0.0079 (6)	−0.0037 (5)
O3	0.0980 (10)	0.0342 (6)	0.0363 (6)	0.0073 (6)	0.0106 (6)	0.0038 (5)
C1	0.0502 (9)	0.0365 (8)	0.0309 (7)	0.0008 (6)	0.0040 (6)	0.0032 (6)
C2	0.0561 (9)	0.0357 (8)	0.0363 (8)	−0.0062 (7)	0.0014 (7)	−0.0028 (6)
C3	0.0509 (9)	0.0283 (7)	0.0391 (8)	−0.0019 (6)	0.0081 (7)	0.0025 (6)
C4	0.0440 (8)	0.0316 (7)	0.0320 (7)	0.0046 (6)	0.0091 (6)	0.0012 (6)
C5	0.0520 (9)	0.0325 (8)	0.0339 (8)	−0.0003 (6)	0.0085 (6)	−0.0050 (6)
C6	0.0537 (9)	0.0282 (7)	0.0383 (8)	−0.0022 (6)	0.0106 (7)	0.0016 (6)
C7	0.0554 (9)	0.0357 (8)	0.0343 (8)	0.0052 (7)	0.0123 (7)	−0.0005 (6)
N1	0.0592 (9)	0.0506 (8)	0.0323 (7)	0.0066 (6)	0.0073 (6)	0.0070 (6)
C8	0.0561 (10)	0.0450 (9)	0.0441 (9)	−0.0015 (7)	0.0036 (7)	−0.0009 (7)
C9	0.0550 (10)	0.0417 (9)	0.0454 (9)	−0.0008 (7)	0.0089 (7)	0.0087 (7)
C10	0.0468 (9)	0.0502 (10)	0.0365 (8)	0.0059 (7)	0.0068 (7)	0.0045 (7)
C11	0.0544 (10)	0.0446 (9)	0.0440 (9)	−0.0011 (7)	0.0057 (7)	−0.0018 (7)
C12	0.0563 (10)	0.0415 (9)	0.0474 (9)	0.0016 (7)	0.0138 (8)	0.0070 (7)
C13	0.0898 (15)	0.0731 (13)	0.0400 (10)	0.0027 (11)	0.0039 (9)	0.0092 (9)

### Geometric parameters (Å, º)

**Table:**

O1—C1	1.3599 (18)	N1—C8	1.329 (2)
O1—H1	0.8200	N1—C12	1.331 (2)
O2—C7	1.2255 (19)	N1—H1A	0.8600
O3—C7	1.2953 (19)	C8—C9	1.369 (2)
C1—C2	1.381 (2)	C8—H8	0.9300
C1—C6	1.389 (2)	C9—C10	1.379 (2)
C2—C3	1.378 (2)	C9—H9	0.9300
C2—H2	0.9300	C10—C11	1.379 (2)
C3—C4	1.384 (2)	C10—C13	1.496 (2)
C3—H3	0.9300	C11—C12	1.370 (2)
C4—C5	1.389 (2)	C11—H11	0.9300
C4—C7	1.487 (2)	C12—H12	0.9300
C5—C6	1.376 (2)	C13—H13A	0.9600
C5—H5	0.9300	C13—H13B	0.9600
C6—H6	0.9300	C13—H13C	0.9600
			
C1—O1—H1	109.5	C8—N1—H1A	120.8
O1—C1—C2	117.94 (13)	C12—N1—H1A	120.8
O1—C1—C6	122.02 (14)	N1—C8—C9	122.28 (16)
C2—C1—C6	120.05 (14)	N1—C8—H8	118.9
C3—C2—C1	119.75 (14)	C9—C8—H8	118.9
C3—C2—H2	120.1	C8—C9—C10	120.04 (15)
C1—C2—H2	120.1	C8—C9—H9	120.0
C2—C3—C4	120.93 (14)	C10—C9—H9	120.0
C2—C3—H3	119.5	C11—C10—C9	117.07 (15)
C4—C3—H3	119.5	C11—C10—C13	121.95 (16)
C3—C4—C5	118.77 (13)	C9—C10—C13	120.97 (16)
C3—C4—C7	121.46 (13)	C12—C11—C10	120.06 (16)
C5—C4—C7	119.74 (13)	C12—C11—H11	120.0
C6—C5—C4	120.85 (14)	C10—C11—H11	120.0
C6—C5—H5	119.6	N1—C12—C11	122.17 (15)
C4—C5—H5	119.6	N1—C12—H12	118.9
C5—C6—C1	119.62 (14)	C11—C12—H12	118.9
C5—C6—H6	120.2	C10—C13—H13A	109.5
C1—C6—H6	120.2	C10—C13—H13B	109.5
O2—C7—O3	122.48 (15)	H13A—C13—H13B	109.5
O2—C7—C4	122.01 (14)	C10—C13—H13C	109.5
O3—C7—C4	115.47 (13)	H13A—C13—H13C	109.5
C8—N1—C12	118.37 (14)	H13B—C13—H13C	109.5
			
O1—C1—C2—C3	178.41 (14)	C5—C4—C7—O2	20.1 (2)
C6—C1—C2—C3	−1.7 (2)	C3—C4—C7—O3	20.0 (2)
C1—C2—C3—C4	1.1 (2)	C5—C4—C7—O3	−157.84 (15)
C2—C3—C4—C5	0.4 (2)	C12—N1—C8—C9	−0.1 (2)
C2—C3—C4—C7	−177.46 (14)	N1—C8—C9—C10	−0.7 (3)
C3—C4—C5—C6	−1.4 (2)	C8—C9—C10—C11	1.2 (2)
C7—C4—C5—C6	176.54 (14)	C8—C9—C10—C13	−178.06 (16)
C4—C5—C6—C1	0.8 (2)	C9—C10—C11—C12	−0.9 (2)
O1—C1—C6—C5	−179.34 (14)	C13—C10—C11—C12	178.39 (17)
C2—C1—C6—C5	0.7 (2)	C8—N1—C12—C11	0.5 (2)
C3—C4—C7—O2	−162.01 (16)	C10—C11—C12—N1	0.0 (3)

### Hydrogen-bond geometry (Å, º)

**Table:**

*D*—H···*A*	*D*—H	H···*A*	*D*···*A*	*D*—H···*A*
O1—H1···O2^i^	0.82	1.85	2.6707 (19)	176
N1—H1*A*···O3^ii^	0.86	1.73	2.5889 (19)	173
C2—H2···O1^iii^	0.93	2.60	3.485 (2)	160

Symmetry codes: (i) *x*, −*y*+1/2, *z*−1/2; (ii) −*x*+1, −*y*, −*z*+1; (iii) −*x*+1, −*y*, −*z*.
